# ORF6 and ORF61 Expressing MVA Vaccines Impair Early but Not Late Latency in Murine Gammaherpesvirus MHV-68 Infection

**DOI:** 10.3389/fimmu.2019.02984

**Published:** 2019-12-18

**Authors:** Baila Samreen, Sha Tao, Karsten Tischer, Heiko Adler, Ingo Drexler

**Affiliations:** ^1^Institute for Virology, Düsseldorf University Hospital, Heinrich-Heine-University, Düsseldorf, Germany; ^2^Department of Oncology-Pathology, Science for Life Laboratory, Karolinska University Hospital, Stockholm, Sweden; ^3^Fachbereich Veterinärmedizin, Institut für Virologie, Freie Universität Berlin, Berlin, Germany; ^4^Comprehensive Pneumology Center, Research Unit Lung Repair and Regeneration, Helmholtz Zentrum Muenchen, German Research Center for Environmental Health (GmbH), Member of the German Center of Lung Research (DZL), Munich, Germany

**Keywords:** vaccinia virus, MVA, T cell response, viral vector vaccine, MHV-68, gammaherpesvirus

## Abstract

Gammaherpesviruses (γHV) are important pathogens causing persistent infections which lead to several malignancies in immunocompromised patients. Murine γHV 68 (MHV-68), a homolog to human EBV and KSHV, has been employed as a classical pathogen to investigate the molecular pathogenicity of γHV infections. γHV express distinct antigens during lytic or latent infection and antigen-specific T cells have a significant role in controlling the acute and latent viral infection, although the quality of anti-viral T cell responses required for protective immunity is not well-understood. We have generated recombinant modified vaccinia virus Ankara (recMVA) vaccines via MVA-BAC homologous recombination technology expressing MHV-68 ORF6 and ORF61 antigens encoding both MHC class I and II-restricted epitopes. After vaccination, we examined T cell responses before and after MHV-68 infection to determine their involvement in latent virus control. We show recognition of recMVA- and MHV-68-infected APC by ORF6 and ORF61 epitope-specific T cell lines *in vitro*. The recMVA vaccines efficiently induced MHV-68-specific CD8+ and CD4+ T cell responses after a single immunization and more pronounced after homologous prime/boost vaccination in mice. Moreover, we exhibit protective capacity of prophylactic recMVA vaccination during early latency at day 17 after intranasal challenge with MHV-68, but failed to protect from latency at day 45. Further T cell analysis indicated that T cell exhaustion was not responsible for the lack of protection by recMVA vaccination in long-term latency at day 45. The data support further efforts aiming at improved vaccine development against γHV infections with special focus on targeting protective CD4+ T cell responses.

## Introduction

Gammaherpesvirus (γHV) infections are life-long and are associated with several oncological and lymphoproliferative disorders particularly after immune suppression in the host (1–3). Severe diseases in humans like Burkitt's lymphoma, Hodgkin's disease, or nasopharyngeal carcinoma are known to be caused by Epstein-Barr virus (EBV). Kaposi's sarcoma, multicentric Castleman's disease or primary effusion lymphoma are related to Kaposi's sarcoma associated herpesvirus (KSHV) (4–9). Primarily, γHV infection of the mucosal epithelium of the naive host is typically asymptomatic. A subsequent productive lytic replication phase allows the formation of latency reservoirs in B cells, dendritic cells and macrophages (10–12). In a suppressed immune system like in AIDS and transplant patients, the reactivation of latent γHV leads to replication followed by disease. However, human γHV are host-specific, therefore studies on vaccine development and strategies against human γHV infection are limited.

Murine γHV 68 (MHV-68) is a natural rodent pathogen that has extensive pathological similarities with and is genetically homologous to human γHV as EBV or KSHV (13, 14). Currently a surrogate model MHV-68 is engaged to understand the pathogenicity of γHV and to examine the efficacy of vaccination strategies. To date several vaccines including attenuated MHV-68, peptide-pulsed DCs, subunit or recombinant DNA vaccines targeting lytic and/or latent viral antigens have shown reduction of acute infections in lungs but were unsuccessful to affect the establishment of latent infection and host vulnerability to viral tumorigenesis (15–22).

Notably, T cells specific for a wide range of epitopes are fundamental to control MHV-68 infection and display two kinetic pattern for acute or latent MHV-68 infection (23). After mucosal infection, CD8+ T cells diminish the load of infectious virus in lungs, while the primary lytic replication seems to have less impact on the latent viral reservoir (24–26). Since MHC class-II positive B cells represent the major latent virus reservoir, cytotoxic CD4+ T cells play a significant role in controlling γHV infection (27). Interestingly, MHV-68 ORF6 (single-stranded DNA binding protein) and ORF61 antigens (ribonucleotide-reductase large subunit protein) are known to stimulate both CD8+ and CD4+ T cells in C57BL/6 mice. ORF6_487_ epitope-specific CD8+ T cells predominantly control early infection while ORF61_524_ epitope-specific CD8+ T cells expand in early latency and circulate at high levels throughout the latent infection period (28–30). Moreover, control of MHV-68 infection is provided by CD4+ T cells specific for ORF6_593_, ORF61_343_, and ORF61_691_ epitopes (31).

Modified vaccinia virus Ankara (MVA) is an attenuated strain of vaccinia virus. Due to the strong clinical safety record MVA has been utilized as a viral vaccine vector targeting recombinant antigens in order to induce T cell responses in immunotherapeutic approaches against cancer and infectious diseases (32–34). We generated recombinant MVA (recMVA) vaccines stably expressing full length MHV-68 ORF6 and ORF61 genes. We choose *en passant* recombineering for insertion of the transgene expression cassette into a self-excisable bacterial artificial chromosome (BAC) containing the MVA genome and allowing for the removal of the selection marker in bacteria (35, 36). Following the rescue of infectious MVA from the self-excisable MVA-BAC, the BAC cassette is efficiently removed from the viral genome resulting in markerless infectious virus progeny. To date, vector vaccine strategies based on recombinant target gene expression were able to control lytic but not latent MHV-68 infection proficiently. Our data show that MVA-based vaccines expressing MHV-68 antigens ORF6 and ORF61 were immunogenic and induced strong CD8+ and CD4+ T cell responses. MVA-ORF6 and MVA-ORF61 proved to be effective in a prophylactic MHV-68 challenge model and were able to protect from MHV-68 early latency by significantly reducing the latent virus reservoir. However, the homologous prime/boost approach failed to protect from latency during the later course of infection despite the presence of antigen-specific CD8+ T cells in high frequencies.

## Materials and Methods

### Cell Lines and Viruses

DF-1 (ATCC CRL 12203), HeLa (ATCC CCL-2), NIH3T3 cells (ATCC CRL 1658), EL4 cells (ATCC TIB-39), and DC2.4 cells (a kind gift of Kenneth L. Rock, University of Massachusetts, USA) were grown in RPMI 1640 supplemented with 10% fetal calf serum (FCS), 100 U/mL penicillin/streptomycin. BHK-21 (ATCC CCL-10) cells were grown in RPMI 1640 supplemented with 5% FCS, 5% tryptose phosphate broth, 100 U/mL penicillin/streptomycin. For bone marrow-derived dendritic cells (BMDCs), bone marrow was collected from tibiae and femurs of C57BL/6 mice. Cells were grown in RPMI 1640 containing 10% FCS, 100 U/mL penicillin/streptomycin and 10% granulocyte-macrophage colony-stimulating factor (GM-CSF) described as previously (37). Working stocks of MHV-68 were prepared by infection of BHK-21 cells as described previously (38). MVA (cloned isolate F6) at 582nd passage on chicken embryo fibroblasts (CEF) was routinely propagated and titered following standard methodology (39).

### Peptides

MHV-68 specific (ORF6_487−495_, ORF61_524−531_, ORF6_593−607_, ORF61_343−357_, ORF61_691−705_) and control peptides (OVA_265−280_, B5_46−60_, βgal_96−103_, and B8_20_) were produced by peptides & elephants GmbH (Hennigsdorf, Germany). Peptides were dissolved in dimethyl sulfoxide (DMSO) at a stock concentration of 1 μg/μl.

### Plasmid Construction

In order to generate MVA transfer plasmids encoding ORF6 or ORF61 MHV-68 genes, respective DNA sequences were PCR amplified by using modified primers designed to generate full length cDNAs of ORF6 and ORF61 including a HA tag sequence at the C-terminal end of each transgene. The cDNAs were cloned in MVA transfer plasmid PH5-dVI-MVA by utilizing *Bam*HI+*Afl*II restriction sites. The expression of both, ORF6 and ORF61, was under the control of the modified PH5-vaccinia virus strong early and late promoter. The linearized transgene expression cassette was excised by *Pac*I restriction enzyme and recombined in deletion VI region of MVA by utilizing 50 bp homologous flanking regions following a two step red-recombination protocol in GS1783 *E. coli* harboring the GFP-expressing MVA-BAC genome resulting in a recMVA-BAC as described previously (40).

### Reconstitution of Recombinant MVA

Rescue of recMVA from BAC was done in DF-1 cells (41). After transfection of recMVA-BAC DNA using turbofect according to the manufacture's protocol (Thermo scientific), rabbit fibroma virus (RFV) (MOI 0.1) was added as helper virus to the cell monolayer. After 72 h, viral plaques (CPE) were monitored by GFP fluorescence. Cells were harvested and pelleted at 4,000 rpm for 10 min at 4°C. Supernatant was discarded and cells resuspended in 1 ml DMEM containing 10% FCS followed by three times freeze-thawing and ultra sonification for 30 s. Supernatant was stored at −80°C. BAC cassette free recMVAs were further identified by limiting dilution on DF-1 cells performed in a 96-well plate. Wild-type MVA-F6, MVA-ORF6, and MVA-ORF61 viruses were propagated and titrated by determining the 50% tissue culture infectious dose (TCID50) in CEF- (39). All viruses were purified by two consecutive ultracentrifugation steps through a 36% (wt/vol) sucrose cushion. Recombinant MVAs were characterized for recombinant ORF6 and ORF61 protein synthesis by western blotting by using monoclonal anti-HA antibody (Sigma) and for replication capacity by *in vitro* low-multiplicity growth kinetics as previously described (42). Briefly, confluent monolayers from one well of six-well tissue culture plates were used per time point. After virus adsorption, the inoculum was removed, cells were washed and further incubated with fresh medium. At multiple time-points post-infection (p.i.), infected cells were harvested and virus was released by freeze±thawing and brief sonication. Serial dilutions of the resulting lysates were plated on confluent CEF monolayers grown in 96-well plates as replicates of eight. At day 7, microscopic analysis monitoring for wells containing viral plaques (CPE) allowed the determination of virus titers by end point dilution as TCID50/ml.

### Generation of T Cell Lines

All T cell lines were established by peptide stimulation of splenocytes obtained from vaccinated mice and maintained by periodical restimulation. For the generation of the CD8+ T cell lines, C57BL/6 mice were vaccinated intraperitoneally (i.p.) once with MVA-ORF6 and MVA-ORF61, respectively, and sacrificed at 8 dpi. For the first round of stimulation, lipopolysacharide (LPS)-activated B cells (LPS-blasts) were generated from splenocytes from naive mice treated with 25 μg/ml LPS and 7 μg/ml dextransulfate for 3 days at 37°C, 5% CO2, and 90% humidity. LPS-blasts were irradiated (30 Gy), pulsed with peptide (250 ng/ml) for 30 min at 37°C and cocultivated with 7 × 10^6^ splenocytes from MVA-ORF6 or MVA-ORF61 vaccinated mice per well in 24-well plates with RPMI 1640 containing 10% FCS, 100 U/ml penicillin, and 100 μg/ml streptomycin for 7 days. For maintenance of T cell lines, the cultures were restimulated every 7 days according to the following scheme. Irradiated (100 Gy), peptide-pulsed (1 μg/ml for 30 min at 37°C) EL4 cells were adjusted to 1 × 10^6^ cells/ml. Irradiated (30 Gy) splenocytes from naive mice were adjusted to 12 × 10^6^ cells/ml. T cells were adjusted to 5 × 10^5^ cells/ml. Finally, 0.5 ml of peptide pulsed EL-4 cells, 0.5 ml of splenocytes, 0.5 ml of 5% TCGF [conditioned medium as supernatant from rat splenocytes stimulated with 5 μg/ml concanavalin A; (37)] and 0.5 ml CD8+ T cells were added in one well of 24 well plate and cocultivated. In order to generate CD4+ T cell lines, C57BL/6 mice were vaccinated twice i.p. with recMVA in a short term prime-boost regimen (prime day 0, boost day 5) as recently shown for CD8 + T cells (43) and sacrificed at 6 d post boost. Spleens were processed as described earlier (37). For maintenance, CD4+ T cell lines were restimulated every 7 days for 20 weeks and thereafter every 14 days as described (37).

### *In vitro* T Cell Assays (T Cell Lines)

BMDCs were infected in 6-well plates with MVA-F6 and recMVA (MOI 5) for 15 h, and MHV-68 (MOI 10) for 48 h. Infected BMDCs 4 × 10^5^/100 μl were incubated with 100 μl of Brefeldin-A (1 μg/ml) and co-cultured with 2 × 10^5^/100 μl of antigen-specific T cells in RPMI 1640 medium supplemented with 10% FCS, 100 U/mL penicillin/streptomycin at 37°C. For peptide-pulsed control cells, 100 μl of peptide were used for CD8+ T cell assays at 1ng/ml and at 1000 ng/ml peptide for CD4+ T cell assays. Briefly, BMDCs were incubated for 30 min at 37°C in the respective peptide solutions, washed and then resuspended in 100 μl of Brefeldin-A (1 μg/ml) and co-cultured with 2 × 10^5^/100 μl of antigen-specific T cells. Intracellular cytokine staining was carried out after 4 h incubation with CD8+ T cells or 15 h with CD4+ T cells as published earlier (37).

### Immunization of Mice

Mouse husbandry was conducted under specific-pathogen-free conditions according to the Federation of European Laboratory Animal Science Associations protocols (FELASA) at the “Zentrale Einrichtung für Tierforschung und wissenschaftliche Tierschutzaufgaben (ZETT).” Experiments were performed in accordance with German animal care and ethics legislation and with the approval of the responsible animal welfare authority. C57BL/6N (6–8 weeks old) female mice were purchased from Janvier. Mice were vaccinated intraperitoneally (i.p) with 1 × 10^8^ IU wild type MVA-F6, MVA-ORF6, or MVA-ORF61 in 200 μl PBS or PBS only as control. Vaccination was performed either as prime only or in a short term prime-boost regimen with a boost i.p. at day 5 (43). Spleens were harvested at day 8 after prime or day 6 after prime-boost to measure T cell responses by intracellular cytokine staining (ICS).

### *Ex vivo* T Cell Analysis (Splenocytes)

For *ex vivo* CD8+ T cell analysis, **s**pleens were removed from vaccinated mice and homogenized with a syringe plunger over metal grid with cell culture medium. Erythrocytes were lysed with 3 ml TAC buffer and washed. Cells were filtered by 70 μm cell strainer and counted. For a short T cell restimulation, 4 × 10^6^ splenocytes were further incubated with respective peptides (1 μg/ml) for 4 h for CD8+ T cells and for 15 h for CD4+ T cells in the presence of BFA. As a control, T cells were stimulated in a non-antigen-specific manner using anti-mouse CD3e antibody (clone 500A2, BD Pharmingen 553238) at 1.25 μg/ml for CD8+ T cells for 4 h and for CD4+ T cells for 15 h in presence of BFA.

### MHV-68 Challenge

To analyze the protective capacity of MVA-ORF6 and MVA-ORF61, mice were prime-boost immunized i.p. with 1 × 10^8^ IU of either recMVA in 200 μl PBS or MVA-F6 or PBS only as controls. Four weeks after boosting, mice were anesthetized and infected intranasally with 5 × 10^4^ PFU of MHV-68 in 20 μl RPMI containing 5% FCS. Mice were sacrificed at day 17 or day 45 after challenge. Spleens were harvested to measure weight and then processed for *ex vivo* T cell analysis (ICS) or used for viral genomic load measurements by qPCR or virus reactivation assays.

### Intracellular Cytokine Staining (ICS)

T cell co-cultures with either infected BMDCs [see section *In vitro* T Cell Assays (T Cell Lines)] or from *ex vivo* peptide-stimulated splenocytes [see section *Ex vivo* T Cell Analysis (Splenocytes)] were transferred into 96-well V-bottom plates. Cells were incubated with blocking buffer [PBS having 1% bovine serum albumin (BSA)] containing 1 μg/ml ethidium monoazide bromide (Life technologies GmbH, Germany) for 20 min on ice under light exposure for live/dead discrimination. Afterwards, ICS was performed using BD Cytofix/Cytoperm fixation/permeabilization kit according to the manufacturer's protocol (BD Pharmingen, Germany). Briefly, cells were washed twice with blocking buffer and surface stained with anti-CD8-PB or anti-CD4-PB for 30 min on ice. In addition, cells were stained for CD62L (L-selectin) in order to discriminate between T cells that were specifically reactivated after *ex vivo* antigen restimulation (CD62L negative) and those that were not reactivated in an antigen-specific manner (CD62L positive). Cells were washed and permeabilized with Cytofix/Cytoperm solution for 15 min on ice. Thereafter, cells were washed, incubated with anti-IFNγ, anti-TNFα or anti-IL-2 for 30 min, washed again, fixed with 2% paraformaldehyde (PFA) and subjected to flow cytometry [BD FACSCanto II (BD Bio Sciences, Germany)].

### *Ex vivo* Reactivation Assay (CPE)

To analyze the frequency of cells carrying viruses reactivating from latency, NIH3T3 cells (10^4^ cell/well) were seeded in 96-well plate. Three-fold dilutions of splenocytes (starting from 1.5 × 10^5^ cells/well) were plated onto NIH3T3 cells with 24 wells per dilution. After 7–14 days, each well was scored for cytopathic effects (CPE) and the frequency of reactivating cells was calculated on the basis of the Poisson distribution by determining the number of cells at which 63.2% of wells scored positive for CPE.

### Measurement of Latent Viral Load by Quantitative Real-Time PCR

Measurement of latent viral load in splenocytes of infected mice was determined by quantitative real-time PCR using the ABI 7300 Real Time PCR System (Applied Biosystems, Foster City, CA) as described previously (44). Briefly, a 70-bp region of the MHV-68 glycoprotein B (gB) gene was amplified, and viral DNA copy numbers were quantified. The murine ribosomal protein L8 (rpl8) was amplified in parallel and used to normalize for input DNA between samples. The data are presented as viral genome copy numbers relative to the copy number of L8.

### Analysis of Exhaustion Markers

Mice were prime/boost immunized and challenged as described in sections Immunization of Mice and MHV-68 Challenge, respectively. Spleens were removed from vaccinated mice and homogenized with a syringe plunger over metal grid with cell culture medium. Erythrocytes were lysed with 3 ml TAC buffer and washed. Cells were filtered by 70 μm cell strainer, counted and stained with aqua dye (Invitrogen) followed by mouse anti-CD8 PB, anti-CD4 PerCP (eBioscience), anti-PD-1 FITC and anti-CTLA-4 APC (Invitrogen) surface antibody staining as well as anti-Eomes PECy7 and anti-T-bet PE (Invitrogen) intracellular antibody staining as described in section Intracellular Cytokine Staining (ICS).

### Statistical Analysis

All data are shown as mean ± SEM of the number of individual mice indicated with n either pooled from or representative for the respective number of independent experiments. The statistical significance was analyzed by an unpaired Student's *t*-test (two-tailed) using GraphPad Prism 6. *p* ≤ 0.05 were considered as significant Manuscript Formatting.

## Results

### Generation and *in vitro* Characterization of Recombinant MVA Vaccines

**Figure 1:**
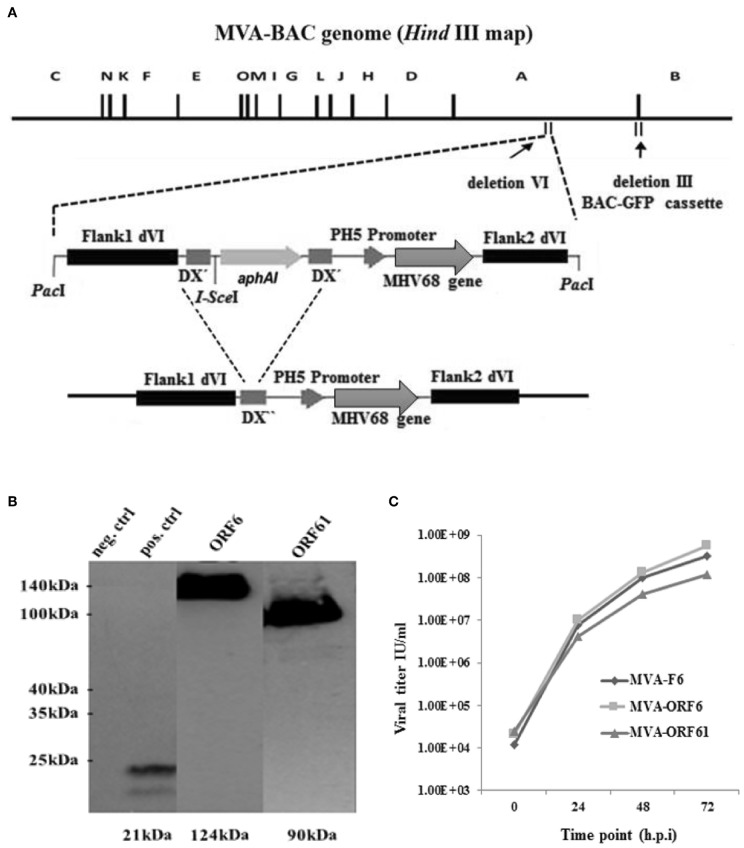
**(A)** Schematic map of the MVA-BAC genome (*Hind*III restriction map) including the BAC-GFP cassette as well as the MVA transfer plasmid containing the respective MHV-68 gene (either ORF6 or ORF61). Flank 1 and 2 are homologous sequences necessary to integrate into the site of deletion IV within the MVA genome. *I-Sce*I restriction site is next to *aph*AI gene, whereas DX″ marks homologous flanks allowing the deletion of *aph*AI through homologous recombination. The final recombinant MVA with *aph*AI marker gene deletion is shown at the bottom. **(B)** Synthesis of recombinant HA-tagged ORF6 (single-stranded DNA binding protein) and ORF61 (ribonucleotide-reductase large subunit protein) (Western blot analysis) after infection of Hela cells with MVA-ORF6, MVA-ORF61, or MVA wild type (as negative control) (MOI-10). For positive control, HeLa cells were transfected with plasmid pIRES-eGFP encoding an HA-tagged HCMV US6 gene and infected with MVA wild type. Cell lysates were harvested after 24 h and run through the SDS 10% PAGE. Immunobloting was done by using HA-specific monoclonal antibodies. The image of the blot was spliced and grouped for editorial purposes. **(C)** Low-multiplicity growth of MVA-ORF6 and MVA-ORF61. Viruses showed similar growth kinetics compared to MVA wild type (MVA-F6) on CEF cells infected at low MOI of 0.01. Viral titers were determined by end point dilution at indicated hours post infection (h.p.i.) to obtain a 50% tissue culture infectious dose (TCID50).

Initially, we cloned cDNAs of MHV-68 ORF6 or ORF61 genes into MVA transfer plasmid PH5_dVI_MVA. After the *Pac*I-restricted digestion of transfer plasmids, the linearized transgene expression cassette was transferred into GS1783 *E. coli* cells containing the MVA-BAC genome including a BAC-GFP cassette residing in the deletion-III region (Figure 1A). The *en passant* technique based on the red recombination system (40) was applied to recombine the transgene expression cassette into deletion VI of the MVA-BAC (36, 40, 45, 46) (Figure 1A). Moreover, the *aphAI* (kanamycin) marker gene present in the expression cassette to positively select recMVA-BAC was removed following *en passant* recombination of homologous sequences of the *I-Sce*I homing endonuclease which flanked the *aphAI* gene (Figure 1A). Furthermore, the complete BAC expression cassette was removed by homologous recombination after BAC rescue (self-excising BAC) in DF-1 cells (35, 41, 47). Sequencing assured the right insertion as well as correct orientation and sequence of the recombinant expression cassette for both recMVA. Additionally, we analyzed the target protein synthesis by using MVA-ORF6 and MVA-ORF61 infected HeLa cell lysates. Western blot analysis using HA-specific antibodies able to bind the C-terminal HA-tag of the recombinant ORF6 and ORF61 proteins confirmed the expected size of ORF6 (124 kDa) and ORF61 (90 kDa), respectively (Figure 1B, blot image spliced and grouped). Importantly, both recMVA showed similar replication capacity *in vitro* as compared to wild type MVA-F6 (Figure 1C).

### Primary CD8+ and CD4+ T Cell Responses After recMVA Vaccination

**Figure 2:**
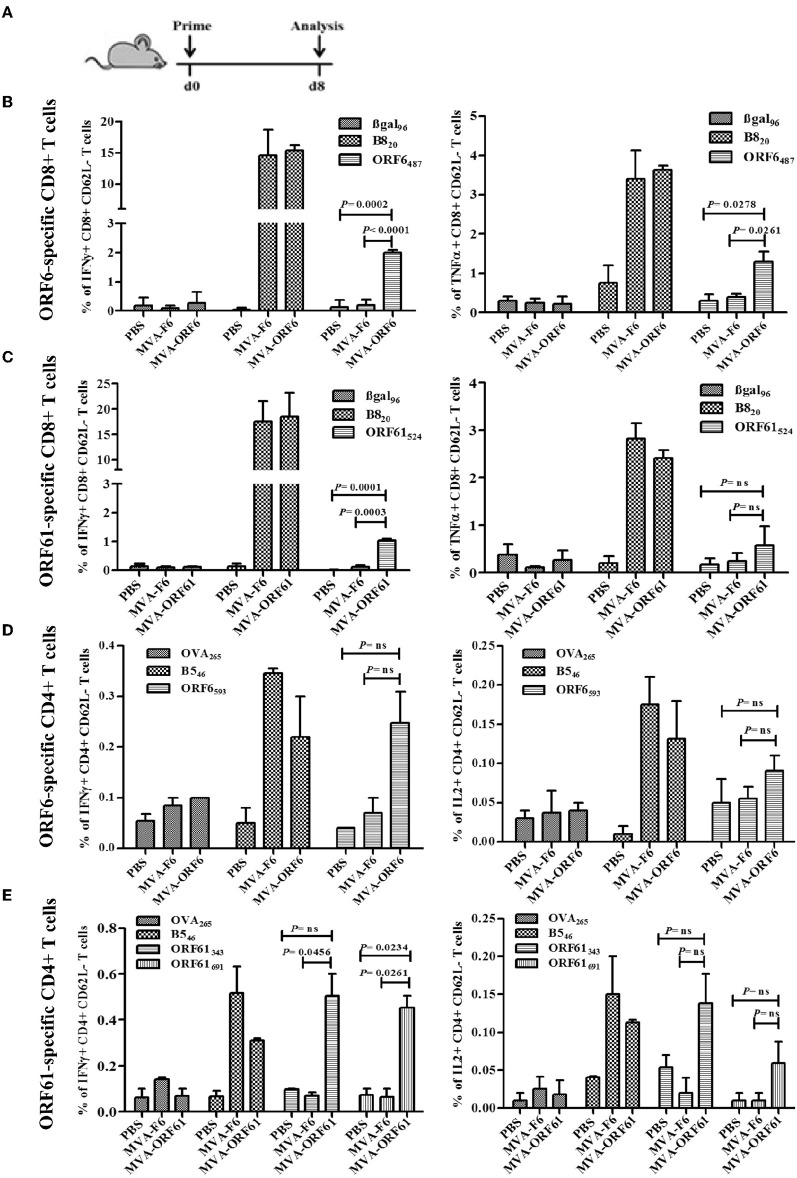
Primary CD8+ and CD4+ T cell response after recMVA vaccination. **(A)** C57BL/6 mice were vaccinated i.p. using 1 × 10^8^ IU/200 μl of MVA-F6 (wildtype), MVA-ORF6 or MVA-ORF61 or 200 μl of PBS as a control group. For *ex vivo* T cell analysis (ICS followed by FACS) splenocytes were prepared from immunized mice at 8 dpi. **(B,C)** CD8+ T cell response. **(B)** Frequencies of activated (CD62L-) ORF6_487_- or **(C)** ORF61_524_ specific CD8+ T cells producing IFNγ (left panel) or TNFα (right panel). βgal_96_ or B8_20_ peptides were used as unrelated or MVA-specific controls, respectively **(D,E)** CD4+ T cell response. **(D)** Frequencies of activated (CD62L-) ORF6_593_- or **(E)** ORF61_343_- and ORF61_691_-specific CD4+ T cells producing IFNγ (left panel) or IL2 (right panel). OVA_265_ or B5_46_ peptides were used as unrelated or MVA-specific controls, respectively. Data shown are mean ± SEM of *n* = 6 mice per group, pooled from 3 **(B,C)** or two independent experiments **(D,E)**.

ORF6- and ORF61-specific CD8+ T cells dominate the MHV-68-directed CD8+ T cell response (25). In addition, ORF6 and ORF61 antigens induce a substantial CD4+ T cell response during MHV-68 infection (31). Thus, we examined the capacity of MVA-ORF6 and MVA-ORF61 vaccines to elicit ORF6- and ORF61-specific CD8+ and CD4+ T cells. T cell responses were determined at day 8 after vaccination (Figure 2A) and showed efficient priming of ORF6- and ORF61-specific CD8+ (Figures 2B,C) and CD4+ T cells (Figures 2D,E) using MVA-ORF6 or MVA-ORF61. About 2% of ORF6_487_-specific and 1% ORF61_524_-specific CD8+ T cells produced IFNγ and to a lesser extend TNFα (Figures 2B,C). ORF6 and ORF61-specific primary CD4+ T cells produced IFNγ and IL-2 after vaccination with MVA-ORF6 or MVA-ORF61, respectively (Figures 2D,E). T-cell responses against MHV-68 antigens accompanied those to dominant MHC class I or II restricted vaccinia virus (VACV) epitopes such as B8_20_ or B5_46_, respectively. B8 and B5 antigens are expressed by various VACV strains including MVA and served as infection controls and helped to validate the efficacy of recMVA vaccination comparative to MVA-wt for CD8+ and CD4+ T cells, respectively (48). Although MVA-ORF6 and MVA-ORF61 were considered to be immunogenic and elicited ORF6- and ORF61-specific CD4+ and CD8+ T cells, some epitope specificities such as ORF6_593_-specific CD4+ T cells were of low frequency after a single vaccination. This was particularly obvious in the memory phase at day 35 p.i. in which CD8+ and CD4+ T cell responses strongly contracted, yet to still detectable levels (Supplementary Figure 1).

### *Ex vivo* Analysis of CD8+ and CD4+ T Cell Responses After Prime-Boost Vaccination

**Figure 3:**
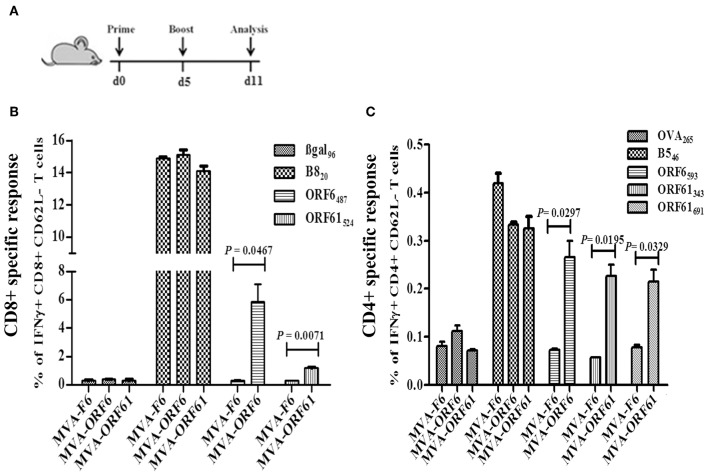
RecMVA prime-boost vaccination **(A)** Vaccination strategy. C57BL/6 mice were vaccinated i.p. with 1 × 10^8^ IU/200 μl MVA-F6, MVA-ORF6 or MVA-ORF61. At day 6 of post boost, splenocytes were prepapared for *ex vivo* T cell analysis (ICS/FACS). **(B)** CD8+ T cells. Frequencies of activated (CD62L-) IFNγ producing ORF6_487_- and ORF61_524_ -specific CD8+ T cells. **(C)** CD4+ T cells. Frequencies of activated (CD62L−) IFNγ producing ORF6_593_−, ORF61_343_−, and ORF61_691_− specific CD4+ T cells. Data shown are mean ± SEM of *n* = 10 mice per group, pooled from two independent experiments.

Next, we determined the antigen-specific T cell responses after prime-boost immunizations with MVA-ORF6 or -ORF61. Mice received a homologous boost vaccination at day 5 post primary immunization (Figure 3A) consistent with a short period prime-boost vaccination protocol (43). This protocol has been demonstrated to result in equally efficient antigen-specific T cell expansion as compared to standard boosting at day 30 post priming. Saving time may be beneficial under certain circumstances in order to allow for earlier protective immunity or to shorten costs. At day 6 post boost, MVA-ORF6 vaccination resulted in higher levels of CD8+ IFNγ+ T cells (6%) specific for ORF6_487_ (Figure 3B) as compared to a single immunization (Figure 2B, Supplementary Figure 1A). The expansion of CD8+ IFNγ+ T cells in the secondary response to MVA-ORF61 (1.5%) was not significant (Figure 3B) compared to a single immunization (Figure 2C, Supplementary Figure 1C). Likewise, high levels of IFNγ producing ORF6_593_-, ORF61_343_-, or ORF61_691_-specific activated CD4+ T cells (0.25–0.3%) were observed (Figure 3C). Interestingly, ORF6_593_-specific responses were similar to the primary response in the acute phase (day 8), whereas ORF61_343_ and ORF61_691_ epitope-specific responses were comparatively reduced (Figures 2D,E). In comparison to the memory response after single immunization, however, we could still observe a boost effect for all epitopes tested (Supplementary Figures 1B,D). The data indicates good capabilities of MVA-ORF6 and -ORF61 to induce antigen-specific memory T cell responses that could reactivate and expand after boost immunization.

### ORF6 and ORF61-Specific T Cell Lines Recognize Infected BMDC *in vitro*

**Figure 4:**
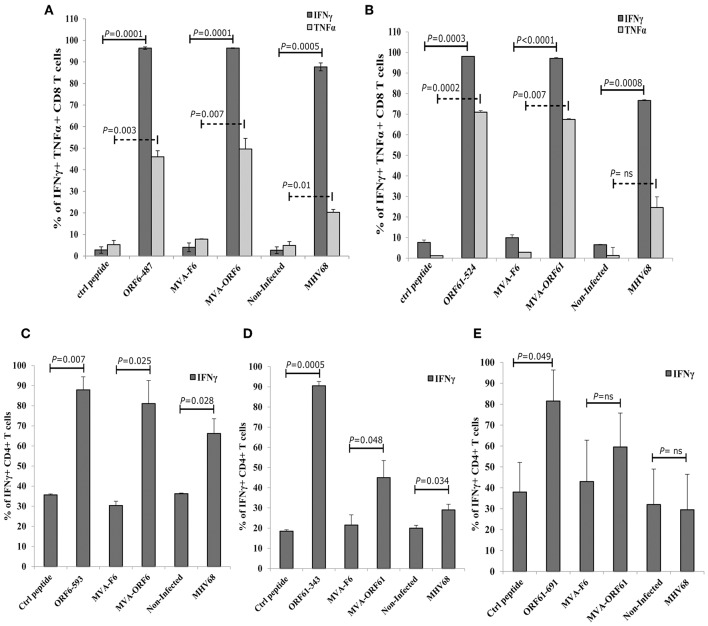
Antigen processing and presentation of ORF6 and ORF61 antigens. After infection with MVA-F6, MVA-ORF6 or MVA-ORF61 (MOI 5) for 15 h or with MHV-68 (MOI 10) for 48 h, BMDCs were co-incubated with CD8+ T cells **(A,B)** or CD4+ T cells **(C–E)**. CD8+ T cells specific for **(A)** ORF6_487_ or **(B)** ORF61_524_ epitopes were co-incubated for 4 h; CD4+ T cells specific for **(C)** ORF6_593_, **(D)** ORF61_343_, or **(E)** ORF61_691_ epitopes for 15 h. BMDCs pulsed with the cognate MHC-class-I **(A,B)** and -II-restricted peptides **(C–E)** at a concentration of 1 or 1,000 ng/ml, respectively, were used as positive controls: **(A)** ORF6-_487_, **(B)** ORF61−_524_, **(C)** ORF6-_593_, **(D)** ORF61−_343_, and **(E)** ORF61−_691_. BMDCs pulsed with irrelevant peptide (ctrl peptide) as well as uninfected or MVA-F6-infected BMDCs served as negative or infection controls, respectively. Cytokine production [IFNγ **(A–E)**, TNFα **(A,B)**] was determined by ICS and FACS analysis. Data are mean ± SEM of *n* = 4 mice per group, pooled from two independent experiments.

Since ORF6 and ORF61 encode both, MHC class-I- and II-restricted epitopes, we tested the endogenous presentation of ORF6- and ORF61-specific epitopes in recMVA or MHV-68 infected target cells by determining the recognition by polyclonal epitope-specific CD8+ or CD4+ T cell lines generated from recMVA-vaccinated mice. Interestingly, BMDCs infected with MVA-ORF6, MVA-ORF61 or MHV-68 and co-cultivated with CD8+ T cells specific for ORF6_487_ and ORF61_524_ epitopes, activated both CD8+ T cell lines as good as peptide-pulsed BMDCs used as a positive control (Figures 4A,B). Likewise, we investigated MHC class-II-restricted endogenous recognition of infected BMDCs by MHV-68-specific CD4+ T cells (Figures 4C–E) (37). ORF6_593_-specific CD4+ T cells were activated indicating efficient MHC class II-restricted presentation by MVA-ORF6 or MHV-68-infected BMDCs (Figure 4C). While MVA-ORF61-infected BMDCs activated ORF61_343_- and ORF61_691_-specific CD4+ T cells, MHV-68-infected BMDCs failed to stimulate both ORF61-specific CD4+ T cell lines (Figures 4D,E). Of note, we determined a comparable affinity for ORF6_487_ and ORF61_524_-specific CD8+ T cells (Supplementary Figure 2A). In contrast to ORF61-specific CD4+ T cells, ORF6_593_-specific CD4+ T cells displayed higher affinity to their peptide/MHC class II complexes (Supplementary Figure 2B).

### Protection From Early Latency but Not From Established Latency by Prophylactic RecMVA Vaccines After MHV-68 Challenge

**Figure 5:**
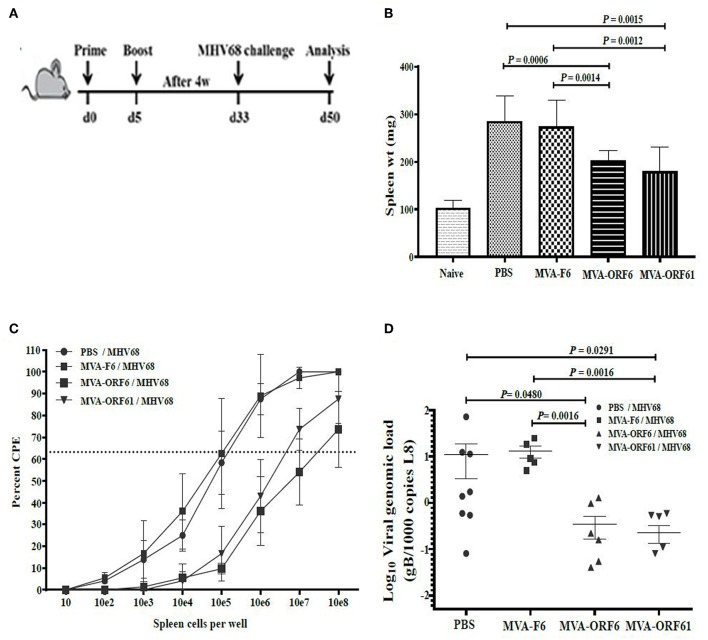
Protection from MHV-68 early latency. **(A)** C57BL/6 mice were i.p. prime/boost vaccinated using 1 × 10^8^ IU/200 μl MVA-ORF6, MVA-ORF61, MVA-F6 (as infection control) or PBS (as mock control). Four weeks later, mice were challenged i.n. by using 5 × 10^4^ PFU/20 μl of MHV-68. At 17 days post challenge, **(B)** spleen weights, and **(C)** latent viral load by *ex vivo* reactivation or **(D)** by Real Time PCR were determined. **(B)** Mean spleen weight ± SEM of n = 8–10 mice per group, pooled from three independent experiments. Age-matched mice which received no treatment at all (no vaccination, no challenge) served as control littermates (naïve, spleen weight ± SEM of *n* = 4). **(C,D)** Single splenocytes suspensions from day 17 after MHV-68 challenge were used **(C)** to determine the latent viral load by *ex vivo* reactivation of splenocytes or **(D)** for isolation of DNA to measure latent viral copy load by Real Time PCR analysis. **(C)** Mean frequency of reactivation replication of latent virus ± SEM from *n* = 6–9 mice per group, pooled from three independent experiments. The horizontal dotted line specifies the point of 63.2% Poisson distribution, set by the non-linear regression that was used to calculate the frequency within the cells. For calculation of significance, frequencies of reactivation events were statistically analyzed by paired *t*-test over all cell dilutions. The statistical difference of MVA-ORF6 immunized group to PBS is *P* = 0.008 and to MVA-F6 *P* = 0.005. The statistical difference of MVA-ORF61 immunized group to PBS is *P* = 0.009 and to MVA-F6 *P* = 0.007. **(D)** Mean viral genomic load in the spleen from *n* = 5–8 mice per group, pooled from two independent experiments is indicated by horizontal bars. Each symbol represents an individual mouse. Statistical significance (*P*).

Following acute infection via the intranasal route, MHV-68 scatter from lungs to secondary lymphoid organs where persistent infection is established in latently infected B-lymphocytes, dendritic cells and macrophages (10). Splenomegaly is a marker for latency as a result of increased B and T cell numbers which peaks at day 14 post infection (24, 49). Accordingly, we determined the ability of recMVA vaccines to protect from viral latency by quantifying the latent viral load and the reactivation capacity of latent viruses (50). We vaccinated mice i.p. followed by a homologous boost vaccination at day 5. Four weeks after the boost, mice were challenged i.n. with MHV-68 (Figure 5A). At day 17 or day 45 post challenge, corresponding to the early or established latent phase of infection, respectively, protection was evaluated.

**Figure 6:**
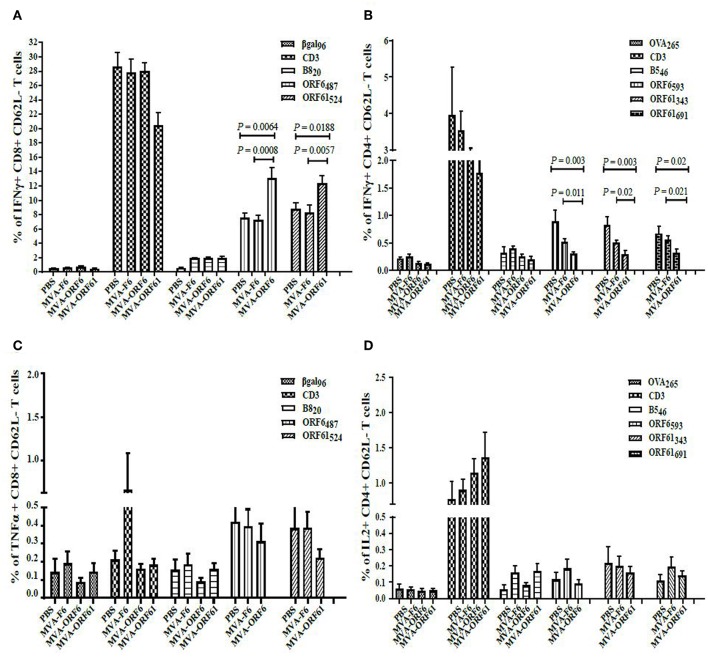
CD8+ and CD4+ T cell responses in recMVA-prime/boost vaccinated mice 17 days after MHV-68 challenge. **(A)** Detection of activated (CD62L−) IFNγ- or **(C)** TNFα-producing ORF6- and ORF61-specific CD8+ T cells (ICS followed by FACS analysis). Splenocytes were stimulated with peptide epitopes ORF6_487_/ORF61_524_, βgal_499_ (negative control) or B8_20_ (vaccination control). **(B)** Detection of activated (CD62L-) IFNγ- or **(D)** IL2-producing ORF6- and ORF61-specific CD4+ T cells. Splenocytes were stimulated with peptide epitopes ORF6_593_, ORF61_343_-, ORF61_691_, OVA_265_ (negative control) or B5_46_ (vaccination control). **(A–D)** CD3 indicates T cell stimulation using anti-CD3 antibodies as non-antigen-specific positive control. The statistical significance was calculated using an un-paired *t*-test. Data shown are mean ± SEM of *n* = 11 mice for each group, pooled from three independent experiments.

At day 17, MVA-ORF6- and MVA-ORF61-pre-immunized mice showed a highly significant reduction in spleen weight (Figure 5B) and a decrease in latent virus as quantified by the number of reactivating splenocytes (Figure 5C) as compared to the control groups (MVA-F6 or PBS). In MVA-F6 or PBS immunized control groups, the frequency of splenocytes allowing for MHV-68 reactivation from latency was 1 in 5,565 cells and 1 in 5,964 cells, respectively. The amount of reactivating splenocytes was 1 in 64,609 cells MVA-ORF61 and 1 in 117,065 cells for MVA-ORF6 immunized animals. Likewise compared to the control groups, MVA-ORF6 or MVA-ORF61 vaccination resulted in a significant decrease of the splenic latent viral genomic load as quantified by real-time PCR (Figure 5D). These results were corroborated by the IFNγ producing CD8+ T cell frequencies in MVA-ORF6 or MVA-ORF61 vaccinated mice after MHV-68 challenge which were significantly higher (Figure 6A). Interestingly, the amount of IFNγ producing CD4+CD62L- T cells were significantly lower in these two vaccination groups as compared to the controls (MVA-F6 or PBS) (Figure 6B). Since these activated IFNγ producing ORF6- and ORF61-specific CD4+ T cells were also significantly reduced in absolute numbers, we suggest that the decrease was not due to a shift in the relative distribution but rather indicates the consumption of these T cell specificities as effectors for direct killing of virus infected cells (Supplementary Figure 3). There were no obvious differences regarding antigen-specific TNFα production in CD8+ T cells (Figure 6C) or IL-2 production in CD4+ T cells (Figure 6D, Supplementary Figure 3) underlining the importance of IFNγ for protection in early latency.

**Figure 7:**
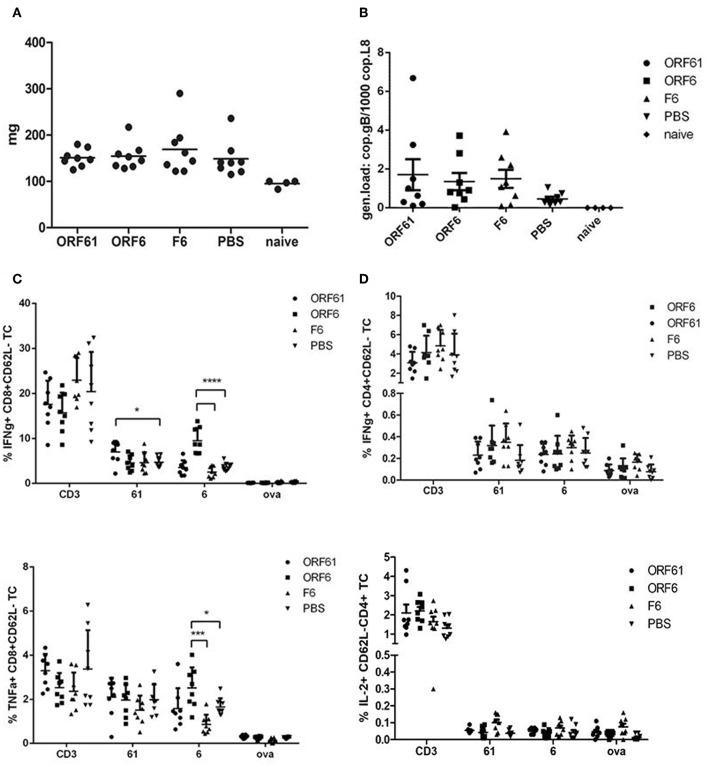
Vaccination with MVA-ORF6 or MVA-ORF61 fails to protect from MHV-68 latency at day 45. C57BL/6 mice were i.p. prime/boost vaccinated using 1 × 10^8^ IU/200 μl MVA-ORF6, MVA-ORF61, MVA-F6 (as infection control) or PBS (as mock control). Four weeks later, mice were challenged i.n. with 5 × 10^4^ PFU/20 μl of MHV-68. Mice which received no treatment at all (no vaccination, no challenge) served as control littermates (naïve). At 45 days post challenge, **(A)** whole spleen weights or **(B)** latent viral copy load by Real Time PCR of DNA isolated from single splenocyte suspensions were determined. **(A)** Mean spleen weight (horizontal bars) of *n* = 4–8 mice per group pooled from two independent experiments. **(B)** Mean viral genomic load in spleen from *n* = 4–8 mice per group pooled from two independent experiments as indicated by horizontal bars. **(C)** CD8+ and **(D)** CD4+ T cell responses in recMVA-prime/boost vaccinated mice 45 days after MHV-68 challenge. **(C)** Detection of activated (CD62L-) IFNγ- (upper panel) or TNFα-producing (lower panel) ORF6- and ORF61-specific CD8+ T cells (ICS followed by FACS analysis). Splenocytes were stimulated with peptide epitopes ORF6_487_, ORF61_524_, or OVA_257_ (negative control). **(D)** Detection of activated (CD62L-) IFNγ- (upper panel) or IL-2-producing (lower panel) ORF6- and ORF61-specific CD4+ T cells. Splenocytes were stimulated with peptide epitopes ORF6_593_, ORF61_343_, or OVA_265_ (negative control). **(C,D)** CD3 indicates T cell stimulation using anti-CD3 antibody as non-antigen-specific positive control. The statistical significance was calculated using an un-paired *t*-test. Data shown are mean ± SEM of *n* = 8 mice for each group, pooled from two independent experiments. Statistical significance (*P*); *P ≤ 0.05; ****P ≤ 0.0001. **(A–D)** Each symbol represents an individual mouse.

Surprisingly, both vaccines failed to confer long-term reduction in latency, since we could not detect a sustained decrease in spleen weight (Figure 7A) and viral genomic load (Figure 7B) at day 45 post challenge as compared to the controls. Interestingly, the T cell analysis showed a significantly higher IFNγ-producing CD8+ T cell response directed against ORF6 and ORF61 as compared to control vaccinated animals (Figure 7C), while CD4+ T cell frequencies for both, ORF6 and ORF61, were comparable in all groups (Figure 7D). In addition, MVA-ORF6 vaccinated mice contained significantly more ORF6-specific TNFα-producing CD8+ T cells compared to the other vaccine groups (Figure 7C, lower panel) which corroborated the data obtained for IFNγ. In contrast, ORF61-specific TNFα-production was comparable between vaccine groups (Figure 7C, lower panel). Interestingly, we failed to detect ORF6- or ORF61-specific IL-2 production in all CD4+ T cells at day 45 (Figure 7D, lower panel) which may indicate that the low CD4+ T cell response seen in long-term latency might be due to lack of proliferation or even exhaustion. Thus, although present in high numbers, antigen-specific CD8+ T cells failed to control latency at later time points.

### Loss of Protection in Late Latency at Day 45 Is Not Driven by T Cell Exhaustion

**Figure 8:**
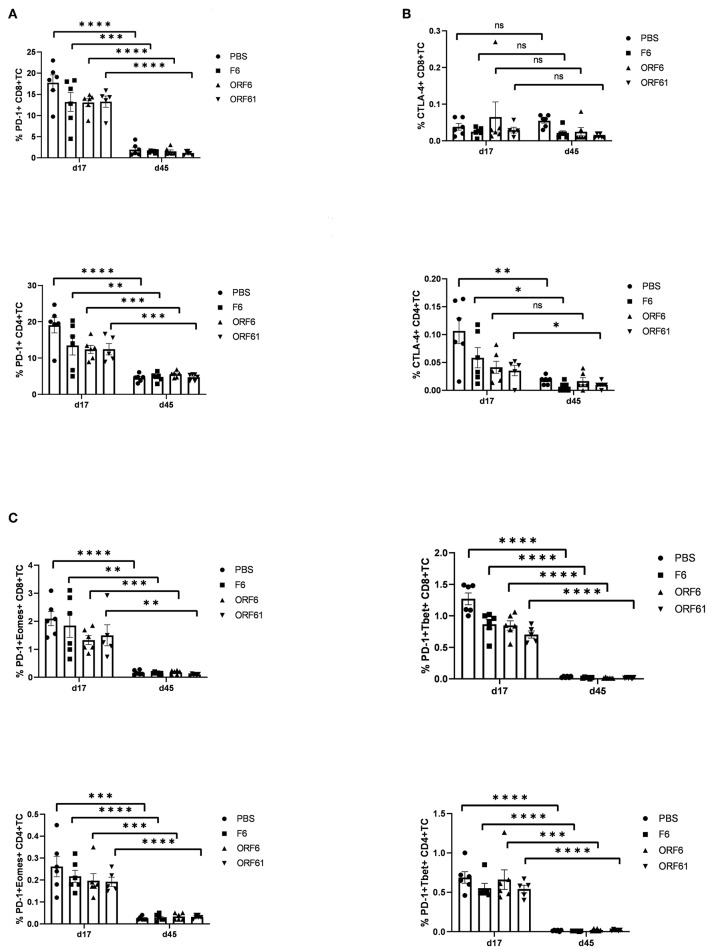
Lack of protection by recMVA vaccines in long-term latency at day 45 post challenge is not due to T cell exhaustion. C57BL/6 mice were i.p prime/boost vaccinated with 1 × 10^8^ IU/200 μl MVA-ORF6, MVA-ORF61, MVA-F6 (as infection control) or PBS (as mock control). Four weeks later, mice were challenged i.n. with 5 × 10^4^ PFU/20 μl MHV-68. On day 17 (d17) or day 45 (d45) post challenge, splenocytes were prepared and the expression of exhaustion markers such as inhibitory receptors PD-1 **(A)** and CTLA-4 **(B)** or transcription factors such as Eomes or T-bet within PD-1-positive T cells **(C)** determined for CD8+ (upper panels) and CD4+ T cells (lower panels) using specific antibodies (FACS analysis). The statistical significance was calculated using an un-paired *t*-test. Data shown are mean ± SEM of *n* = 5–6 mice for each group, pooled from two independent experiments. Statistical significance (*P*); *P ≤ 0.05; **P ≤ 0.01; ***P ≤ 0.001; n.s., Non-significant.

As mentioned above, T cell exhaustion especially targeting CD4+ T cells during the course of MHV-68 infection could be a reason for the transient and timely limited protective capacity of recMVA vaccines. We therefore monitored the CD8+ and CD4+ T cell responses at day 17 and day 45 post challenge for the expression of characteristic markers of T cell exhaustion such as the inhibitory receptors PD-1 and CTLA-4 as well as the transcription factors Eomes and T-bet (Figure 8). Interestingly, the expression of exhaustion marker PD-1 was highly significantly reduced in CD4+ and CD8+ T cells in all groups at day 45 compared to day 17 (Figure 8A). The expression of CTLA-4 did not change between day 45 and day 17 in CD8+ T cells and was significantly reduced in CD4+ T cells for all groups apart from the MVA-ORF6-vaccinated group which showed comparable expression of CTLA-4 at days 45 and 17 (Figure 8B). In addition, PD-1/T-bet double positive cells most likely representing early exhausted T cells as well as PD-1/Eomes double positive cells indicating most likely terminally exhausted T cells were significantly diminished in CD4+ as well as CD8+ T cells in all corresponding groups at day 45 compared to day17 (Figure 8C). Of note, T cell frequencies were considerably low and close to detection limit for all markers except PD-1 at day 45. Since exhaustion markers were not increased in any of the groups by day 45, but in contrast strongly decreased at day 45 with the exception of CTLA-4 in CD8+ T cells, we conclude that T cell exhaustion did not contribute to the loss of protection by recMVA vaccines in established latency at day 45.

## Discussion

Recombinant vaccines based on MVA expressing MHV-68 antigens have been constructed employing an MVA-BAC recombination system based on the *en passant* technique which has been described as a comparatively fast and easy method (35). In favor of possible vaccine application, we applied a self-excisable MVA-BAC in which the BAC cassette was subsequently removed from recMVA genomes by site specific recombination (41). BAC-less MVA-ORF6 and MVA-ORF61 efficiently expressed ther recombinant target genes and displayed standard growth kinetics *in vitro*.

As T cells are pivotal to control γHV infections, we monitored the T cell response after immunization with recMVA and investigated the efficacy of MHV-68-infected target cells to stimulate CD8+ and CD4+ T cells *in vitro*, as an indication of direct antigen processing and presentation. Nevertheless, direct as well as cross-priming contribute to MVA-induced CD8+ T cell responses (51–53). After a single vaccination with MVA-ORF6 or MVA-ORF61 we observed strong but subdominant target antigen-specific effector CD8+ T cell responses during the acute phase of infection (8 dpi) (43, 54–56). As expected, in the memory phase of infection CD8+ T cell frequencies contracted to about 10% of the initial maximum (Supplementary Figures 1A,C). ORF6 and−61 antigens were expressed under control of the modified PH5 early/late promoter resulting in enhanced transgene expression (57). In addition, both MVA-ORF6 and MVA-ORF61 vaccines induced effector CD4+ T cell responses upon primary vaccination, but only ORF6-specific CD4+ T cells were clearly detectable in the memory phase (35 dpi) (Supplementary Figures 1B,D). Of note, next to ORF61-specific memory CD4+ T cells, MVA-ORF61-induced B5_46_-specific memory CD4+ T cell responses were hardly detectable as well, implicating that these CD4+ T cells primarily differentiated into effector T cells (CD62L^low^) after immunization leaving low memory CD4+ T cell responses (58). RecMVA has been often used as vector for boost vaccinations (59–61) and allows for short-term prime-boost immunization strategies (43). Likewise, our data indicate that MVA-ORF6− and -ORF61 efficiently expanded CD4+ and CD8+ T cells following this short-term prime-boost regimen even at low to undetectable numbers of pre-existing memory CD4+ T cells.

Furthermore, the endogenous processing and presentation of viral antigens by infected target cells was analyzed by using antigen-specific CD8+/CD4+ T cell lines which were generated from recMVA-vaccinated mice. These effector memory-like T cells were used as *in vitro* readout system (37, 43). MHV-68 ORF6 which encodes for a single-stranded DNA binding protein, seemed to be efficiently processed and both, MHC-I- and -II restricted epitopes, presented as ORF6-specific CD8+ and CD4+ T cells were activated upon exposure to MVA-ORF6 or MHV-68-infected target cells. After infection of target cells with recMVA or MHV-68 encoding ORF61 a ribonuleotide-reductase large subunit protein, CD8+ T cells were specifically activated, while CD4 T cells were less responsive (recMVA) or completely failed to respond (MHV-68). Interestingly, EBV-BPLF1 is known to interact with its large subunit with ribonucleotide-reductase and removes its ubiquitin chain, thereby decreasing the activity of ribonucleotide-reductase *in vitro*, while MHV-68 ribonucleotide-reductase has been shown to colocalize with subnuclear structures named PML-nuclear bodies (promyelocytic leukemia protein) (62). We propose the possibility that processing of ORF61 as an antigen encoding MHC-II-restricted epitopes might be decreased *in vitro* due to the MHV-68 deubiquitinase enzyme (DUB) encoded by ORF64 similar as in the case of EBV. Further studies are required to elucidate the processing of the ORF61 antigen.

Our data indicate that recMVA vaccines induce MHV-68–specific long-term memory T cell responses after primary as well as boost vaccination. Previously, post exposure vaccinations with recombinant replication-competent vaccinia viruses (VACV-ORF6_487_ and VACV-Orf61_524_) have shown a transient viral replication control in lungs but had no impact on latency establishment (28). Therefore, we were interested in the protective efficacy of recMVA on the establishment of MHV-68 latency at early (day 17) and late time points (day 45) in a prophylactic vaccination setting. At day 17 in early latency, the spleen weights and splenic viral copy loads were significantly reduced in both groups of recMVA-immunized mice. However, in established latency at day 45, both vaccination groups were comparable to the controls and the protective capacity was lost. CD8+ T cells are involved in resolving splenomegaly which is important in control of long-term latent infection, while the manifestation of splenomegaly is driven by CD4+ T cells depending on MHV-68-infected B-cells in the spleen (63–67). We observed that both rec-ORF6-MVA and rec-ORF61-MVA vaccines maintained strong effector CD8+ T cell responses for both time points tested in the latent phase of MHV-68 infection. Those antigen specific CD8+ T cells were able to proliferate efficiently in response to a challenge with MHV-68 encoding the cognate epitopes. In contrast, ORF6- and ORF61-specific effector CD4+ T cells were significantly reduced in frequency and absolute numbers as compared to mice without pre-existing immunity to ORF6 or ORF61 in early latency, while they were indistinguishable from the controls at day 45.

CD8+ T cells are not sufficient to prevent lytic or persistent MHV-68 infection, however they contribute to CD4+ T cells which mainly control the latent infection (67). These CD4+ T cells have effector functions which are mediated by IFNγ production and/or cytotoxicity. Our data corroborate this view, since rec-MVA induced strong effector CD4+ T cells producing IFNγ which may have been consumed to control the latent infection. MHV-68 shows persistent infection in IFNγ^−/−^ or IFNγ receptor^−/−^ mice, indicating a role of IFNγ in limiting the lytic infection or inhibiting reactivation from latent infection (68, 69). The loss of ORF6 and ORF61 specific CD4+ T cells in recMVA-vaccinated groups after challenge supports this effector function and correlates with decreased spleen weight as a marker for splenomegaly (63, 70) as well as the reduced latent virus reservoir in these mice early at day 17. However, these CD4+ T cells were unable to control long-term latency and were present at comparable frequency as in control groups, possibly because they were consumed and/or unable to proliferate. The latter is supported by the loss of IL-2 production in these cells at day 45 supporting the view that particularly CD4+ T cells may be required for long-term infection control (27, 71). T cell exhaustion as described for some chronic infections (72) could be a reason for the restricted protective capacity of recMVA vaccines. However, a comparative T cell analysis for exhaustion markers such as the inhibitory receptors PD-1 and CTLA-4 or the transcription factors Eomes and T-bet at day 17 and day 45 excluded a role of T cell exhaustion at day 45. The CD4+ T cell frequencies expressing PD-1 or CTLA-4 were significantly decreased in all corresponding groups at day 45 compared to day 17, with the exception of the MVA-ORF6 group. Similarly, we found a significant decrease of CD4+ and CD8+ T cell frequencies coexpressing exaustion markers such as PD-1 and T-bet (early exhaustion) or PD-1 and Eomes (terminal exhaustion) (72–74) in corresponding groups at day 45 compared to day 17. We therefore conclude that the lack of protection by recMVA vaccines in established latency in this model was not mediated by T cell exhaustion.

Up to now exclusively live-attenuated γHV e.g., latency-deficient mutants were able to partially protect from latency (18, 75–78). A more recent study demonstrated that the protection mediated by latency-deficient mutants does not require immunity to any of the known latency genes in MHV-68. This suggests that lytic access to the latency reservoir is indeed a viable target for control (79). The prime/boost vaccination regimen based on recMVA expressing ORF6 and ORF61 had a profound impact on the quality and quantity of MHV-68-specific T cell responses. The vaccines proved to be protective in early latency by limiting latent viral infection. The failure to protect from long-term latency offers now the chance and basis to test advanced strategies to further enhance the CD4+ T cell response such as heterologous prime/boost regimens by combining antibody-targeted vaccines or DNA-, protein- or viral vector-based (e.g., Adeno, VSV) vaccines for priming and MVA for boosting. These approaches have been shown to significantly increase target antigen-specific T cell responses and enhance their efficacy in preclinical models as well as in clinical studies (80, 81). Alternatively, direct targeting of ORF6 or-61 antigens to the MHC class II processing pathway, e.g., by fusion to the MHC class II-associated invariant chain Ii, may be an option. A recent study provided evidence that combined intranasal/intramuscular vaccination of mice using a heterologous adenovirus-based prime/boost protocol reduced the risk of latency establishment after intranasal MHV-68 challenge. Interestingly, exclusively CD8+ T cells were targeted by the polytope vaccine and it was hypothesized that their redirection to the mucosal viral entry site abrogated the infection before latency was established (82). Alternatively, tissue-resident memory cells may have been induced and efficiently controlled the initial viral challenge load at the mucosa. The recMVA evaluated here seem promising model candidates to test immunotherapeutic approaches for long-term latent virus control even in immunocompromised hosts, e.g., by simultaneous vaccination with both constructs or in combination with adoptive transfer of antigen-specific T cells. Our findings have important implication for the use and the future design of vaccine based immunotherapy against gammaherpesvirus infections.

## Data Availability Statement

All datasets generated for this study are included in the article/Supplementary Material.

## Ethics Statement

The animal study was carried out in accordance with the recommendations of the Society for Laboratory Animal Science (GV-SOLAS) and the European Health Law of the Federation of Laboratory Animal Science Associations (FELASA). The protocol was approved by the North Rhine-Westphalia State Agency for Nature, Environment and Consumer Protection (LANUV), Germany (Permit Numbers: A116/12 and G237/14).

## Author Contributions

BS, ST, KT, HA, and ID conceived and designed the experiments. BS, ST, and HA performed the experiments. BS, ST, and ID analyzed the data. BS and ID wrote the paper.

### Conflict of Interest

The authors declare that the research was conducted in the absence of any commercial or financial relationships that could be construed as a potential conflict of interest.
