# Supplementary material for: ORF6 and ORF61 Expressing MVA Vaccines Impair Early but Not Late Latency in Murine Gammaherpesvirus MHV-68 Infection
Source: Front Immunol. 2019 Dec 18;10:2984. doi: 10.3389/fimmu.2019.02984 (PMC6930802; doi:10.3389/fimmu.2019.02984)
Supplement: Supplementary file 1 [file Data_Sheet_1.pdf]

Supplementary Material

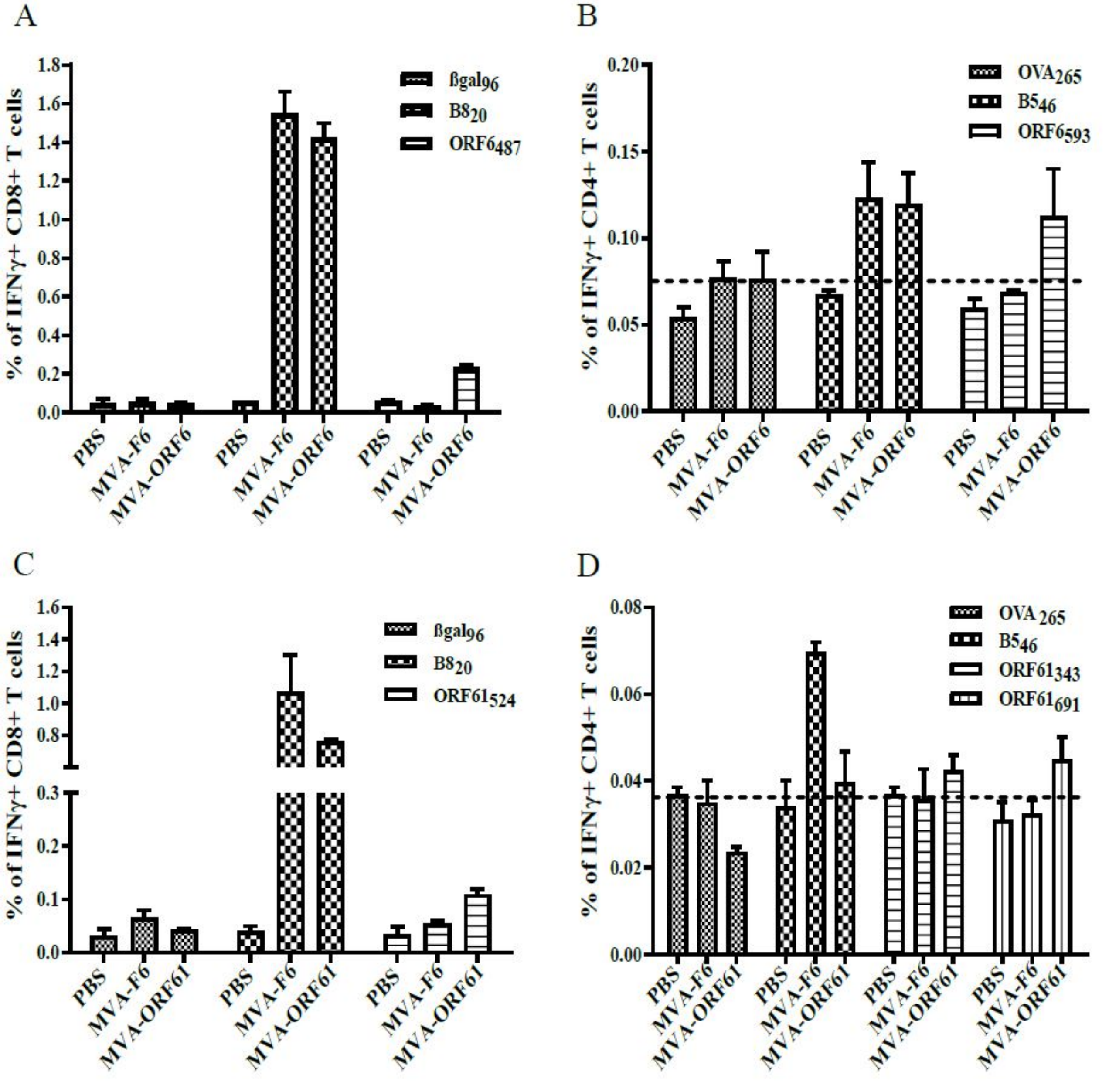

Supplementary Figure 1. Memory CD8+ and CD4+ T cell responses after i.p. vaccination of C57BL/6 mice with 1x10^{8} IU/200µl MVA-F6, MVA-ORF6, MVA-ORF61 or PBS. For *ex vivo* T cell analysis (ICS followed by FACS) splenocytes were prepared from immunized mice at 35 dpi. (A, B) ORF6-specific memory T cells. Frequencies of IFNγ producing CD8+ (**A**) and CD4+ T cells (**B**). (**C, D**) ORF61-specific memory T cells. Frequencies of IFNγ producing CD8+ (**C**) and CD4+ T cells (**D**). (**A, C**) βgal$_{96}$ or B8$_{20}$ peptides as well as OVA$_{265}$ or B5$_{46}$ peptides (**B, D**) were used as unrelated or MVA-specific peptide controls, respectively. Data shown are mean ± SEM of n=6 mice per group, pooled from 2 independent experiments.
