# Supplementary material for: ORF6 and ORF61 Expressing MVA Vaccines Impair Early but Not Late Latency in Murine Gammaherpesvirus MHV-68 Infection
Source: Front Immunol. 2019 Dec 18;10:2984. doi: 10.3389/fimmu.2019.02984 (PMC6930802; doi:10.3389/fimmu.2019.02984)
Supplement: Supplementary file 2 [file Data_Sheet_2.pdf]

**Supplementary Material**

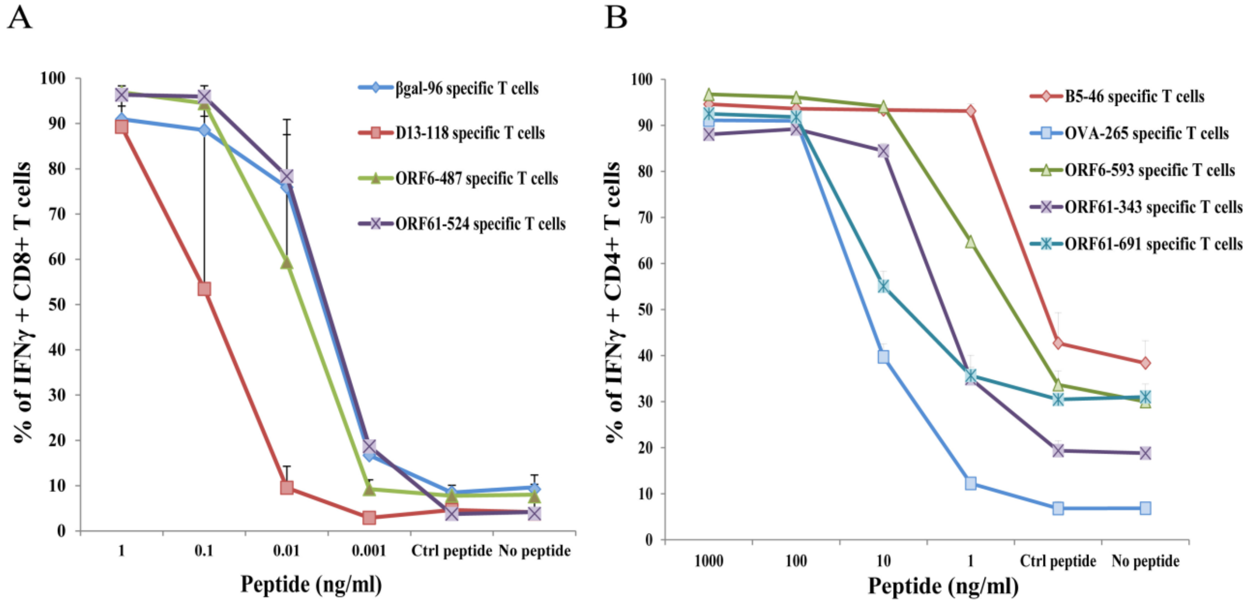


**Supplementary Figure 2.** Avidity of MHV-68 antigen-specific CD8+ and CD4+ T cell lines. (**A**) Avidity of CD8+ T cell lines. DC2.4 cells were pulsed at different peptide concentrations ($10^{-8}$M (1ng/ml) to $10^{-11}$M (0.001ng/ml)). (**B**) Avidity of CD4+ T cell lines. BMDCs were pulsed at different peptide concentrations ($10^{-5}$ M (1000ng/ml) to $10^{-8}$M (1ng/ml)). CD8+ or CD4+ T cells with indicated specificity were added to DC2.4 cells or BMDC's, respectively. Target cells were pulsed with either the cognate peptide at declining concentrations or an irrelevant but MHC-binding control peptide at highest concentration or no peptide. After 4h incubation, frequencies of IFNγ-producing CD8+ (**A**) and CD4+ T cells (**B**) were determined by intracellular cytokine staining (ICS) followed by FACS analysis. Data are mean ± SEM of two independent experiments).
