# Supplementary material for: ORF6 and ORF61 Expressing MVA Vaccines Impair Early but Not Late Latency in Murine Gammaherpesvirus MHV-68 Infection
Source: Front Immunol. 2019 Dec 18;10:2984. doi: 10.3389/fimmu.2019.02984 (PMC6930802; doi:10.3389/fimmu.2019.02984)
Supplement: Supplementary file 3 [file Data_Sheet_3.pdf]

Supplementary Material

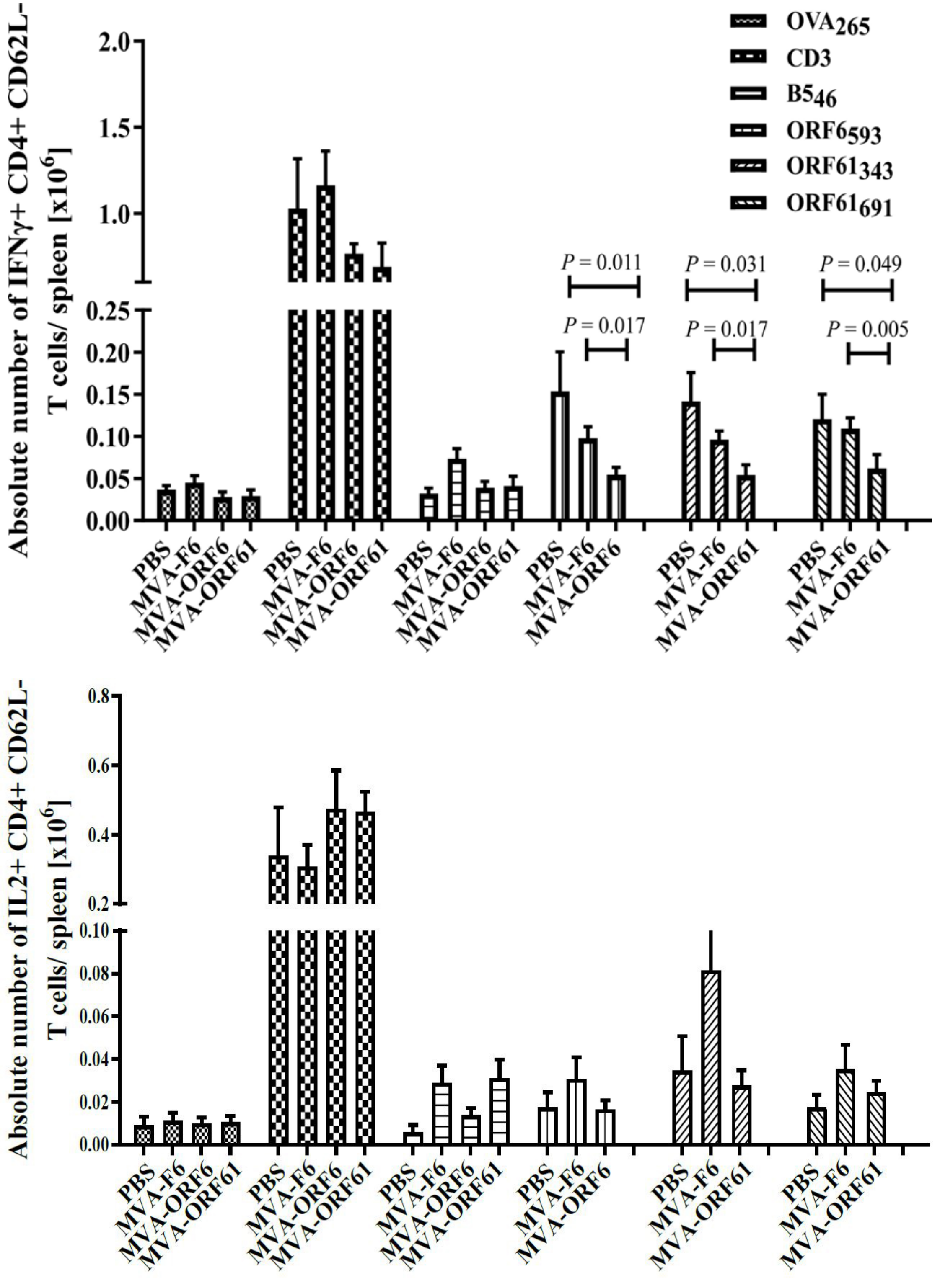

Supplementary Figure 3. *Ex vivo* CD4+ T cell responses after recMVA vaccination followed by MHV-68 challenge (**absolute numbers**). Mice were i.p. prime/boost vaccinated (boost at day 5 post prime) with MVA-F6, MVA-ORF6 or MVA-ORF61 using $1x10^8$ IU/200µl or PBS. Four weeks later, mice were challenged i.n. with MHV-68 ($5x10^4$ PFU/20µl). At day 17 after challenge, splenocytes were subjected to ICS followed by FACS analysis. Absolute numbers of activated (CD62L-) IFNγ- (upper panel) or IL2-producing ORF6- and ORF61-specific CD4+ T cells (lower panel). Splenocytes were stimulated with peptide epitopes $ORF6_{593}$, $ORF61_{343}$, $ORF61_{691,}$ OVA_{265} (negative control) or $B5_{46}$ (vaccination control). CD3 indicates non-antigen-specific T cell stimulation using anti-CD3 antibodies. Data shown are mean ± SEM of n=11 mice per group, pooled from 3 independent experiments.
